# Relationship between Interhemispheric Inhibition and Dexterous Hand Performance in Musicians and Non-musicians

**DOI:** 10.1038/s41598-019-47959-y

**Published:** 2019-08-09

**Authors:** Yi-Ling Kuo, Jason J. Kutch, Beth E. Fisher

**Affiliations:** 1 Division of Biokinesiology and Physical Therapy, University of Southern California, Los Angeles, CA USA; 2 Massachusetts General Hospital Institute of Health Professions, Boston, MA USA; 3 Neuroscience Graduate Program, University of Southern California, Los Angeles, CA USA; 4 Department of Neurology, Keck School of Medicine, University of Southern California, Los Angeles, CA USA

**Keywords:** Motor cortex, Inhibition

## Abstract

Interhemispheric inhibition (IHI) is essential for dexterous motor control. Small previous studies have shown differences in IHI in musicians compared to non-musicians, but it is not clear whether these differences are robustly linked to musical performance. In the largest study to date, we examined IHI and comprehensive measures of dexterous bimanual performance in 72 individuals (36 musicians and 36 non-musicians). Dexterous bimanual performance was quantified by speed, accuracy, and evenness derived from a series of hand tasks. As expected, musicians significantly outperformed non-musicians. Surprisingly, these performance differences could not be simply explained by IHI, as IHI did not significantly differ between musicians and non-musicians. However, canonical correlation analysis revealed a significant relationship between combinations of IHI and performance variables in the musician group. Specifically, we identified that IHI may contribute to the maintenance of evenness regardless of speed, a feature of musical performance that may be driven by practice with a metronome. Therefore, while IHI changes by themselves may not be sufficient to explain superior hand dexterity exhibited by musicians, IHI may be a potential neural correlate for specific features of musical performance.

## Introduction

Musical expertise serves as a distinctive model to study practice-induced neuroplasticity in the context of exceptional bimanual hand coordination. Communication between the two hemispheres is essential for the musicians to acquire high levels of skill in expressing their artistry. However, the translation of musical training-induced brain remodeling to more general skills that require coordinated ***movements*** between the hands has not been determined. There is evidence that musical training could potentially generalize to cognitive development in children (e.g. language, working memory, intelligence) and for maintaining cognitive function in aging adults^1–5^. Although musical performance is a bimanual motor task which is highly trained over years of practice, the long-term impact of musical training on bimanual motor function has not been substantially addressed. Moreover, studies utilized neurophysiological measures to understand the substrates underling bimanual motor control in professional musicians did not comprehensively characterize hand motor ability developed with prolonged intensive instrument playing^6–9^ and were often limited in result generalization due to small sample size. Thus, this study asks whether the interhemispheric interactions and bimanual motor coordination acquired by musicians who have trained intensively with a musical instrument generalizes to other bimanual skills.

Interhemispheric inhibition (IHI) is an essential cortical mechanism underlying most forms of motor control, but is considered a crucial feature of fine dexterous motor control^10–13^. The sophisticated and finely tuned bimanual coordination required of an expert musician offers an unprecedented opportunity to explore human performance limits and to advance our understanding of experience-dependent interhemispheric remodeling. For example, we know that the brain’s inhibitory circuitry has a significant role in the execution of dexterous hand movements and in the performance of tasks with a high skill demand^10,12^. The extraordinary skill level associated with highly trained musicians in a few previous studies appears to demonstrate neurophysiologic evidence that includes greater IHI compared with non-musicians^14–16^. However, it is not known to what degree this enhanced IHI is a task-specific phenomenon associated with playing an instrument or instead a more task-independent phenomenon associated with other motor skills that require some form of coordination between the hands. Given that bimanual coordination may require performance of skills in which both hands spatially do the same thing (i.e. symmetric bimanual coordination) or skills in which each hand performs a different action at the same time (i.e. asymmetric bimanual coordination), both types of bimanual coordination tasks were investigated.

Musical training has been shown to induce neuroplastic changes. The majority of research has focused on either brain differences between musicians and non-musicians without consideration of hand motor control^15,17–19^ or differences between musicians and non-musicians in hand motor control without examination of any brain measures. Additionally, those studies that have evaluated motor function in musicians compared to non-musicians have mostly utilized simple unimanual tasks^9,20–22^. There have been studies measuring brain activity synchronized with movements in musicians using neuroimaging, such as task functional MRI^7,23,24^. However, due to the limited space inside the scanner and no visual feedback from the hands available, motor tasks were often designed with simplicity to ensure its feasibility to perform while lying down in the scanner. The single study that used both IHI and measures of motor function in musicians, investigated the impact of IHI asymmetry on unimanual hand function^16^. It has yet to be determined if changes in IHI in musicians are associated with bimanual motor coordination. Therefore, the current study aimed to investigate the relationship between IHI and bimanual coordination in musicians compared with non-musicians. We hypothesized that a stronger relationship between IHI and bimanual coordination (i.e. both symmetric and asymmetric tasks) would be observed in musicians compared with non-musicians. An alternative hypothesis, however, is that a stronger relationship between IHI and bimanual coordination in musicians will be evident in only one form of bimanual coordination, either a symmetric or asymmetric task, in accordance with the skill acquired from symmetric (e.g. piano) or asymmetric (e.g. violin) instrument training.

## Results

Participants’ demographic data are summarized in Table 1. There was no significant group difference in age [t(_70_) = −1.87, *p* = 0.07, *d* = 0.54] and handedness as quantified by the laterality quotients of the Edinburgh Handedness Inventory [t(_70_) = −0.89, *p* = 0.38, *d* = 0.21]. There were four left-handed and four mix-handed participants in the musician group, as well as three left-handed and three mix-handed participants in the non-musician group. The musicians demonstrated significantly greater musical sophistication as evidenced by the Goldsmiths Musical Sophistication Index questionnaire (*p* < 0.001). Among the 36 musicians, 6 of them played only one instrument, and 13 of them played secondary instruments in the same category as the primary instrument (e.g. double bass as primary instrument, cello as secondary instrument). However, all of the recruited musicians received intensive classical instrument training only in their primary instruments (i.e. have majored in instrument performance in only one musical instrument and spent most of the practicing time on that instrument).

**Table 1:** Demographics in the musician and non-musicians.

	Musicians	Non-musicians	Independent *t* test
Number	36	36	
Age (years)	25.0 ± 7.0	28.0 ± 3.5	t_(70)_ = −1.87, *p* = 0.07, *d* = 0.54
Handedness (laterality quotients, LQ)	54.1 ± 51.4	64.1 ± 43.1	t_(70)_ = −0.89, *p* = 0.38, *d* = 0.21
Musical sophistication (score)			
Active engagement	48.6 ± 4.2	34.8 ± 8.5	t_(51.0)_ = 8.7, *p* < 0.001, *d* = 2.06
Perceptual abilities	43.9 ± 2.5	39.3 ± 4.5	t_(54.4)_ = 5.4, *p* < 0.001, *d* = 1.26
Musical training	33.1 ± 2.8	21.8 ± 5.0	t_(55.2)_ = 12.0, *p* < 0.001, *d* = 2.79
Singing abilities	32.8 ± 3.7	26.0 ± 4.8	t_(70)_ = 6.7, *p* < 0.001, *d* = 1.59
Emotions	32.3 ± 3.4	27.0 ± 4.0	t_(70)_ = 6.0, *p* < 0.001, *d* = 1.43
General musical sophistication	85.6 ± 5.6	66.7 ± 8.9	t_(58.6)_ = 10.8, *p* < 0.001, *d* = 2.54
Training start age (years)	6.5 ± 3.2	NA	
Total training time (years)	18.5 ± 7.4	NA (<1 year)	
Average daily practice time (hours)	3.1 ± 1.3	0 hours	
Total practice time past week (hours)	19 ± 11.8	0 hours	

Values are group means ± SD. Independent *t* tests were used to compare the means of age, handedness, and musical sophistication between musicians and non-musicians. *t* = t-value of independent samples *t* test; *d* = Cohen’s *d* as effect size.

Handedness categories: left handed: LQ < −40; mix handed: −40 ≤ LQ ≤ 40; right handed: LQ > 40.

NA: not available due to incomplete subject report.

### Bimanual coordination outcomes

**Figure 1:**
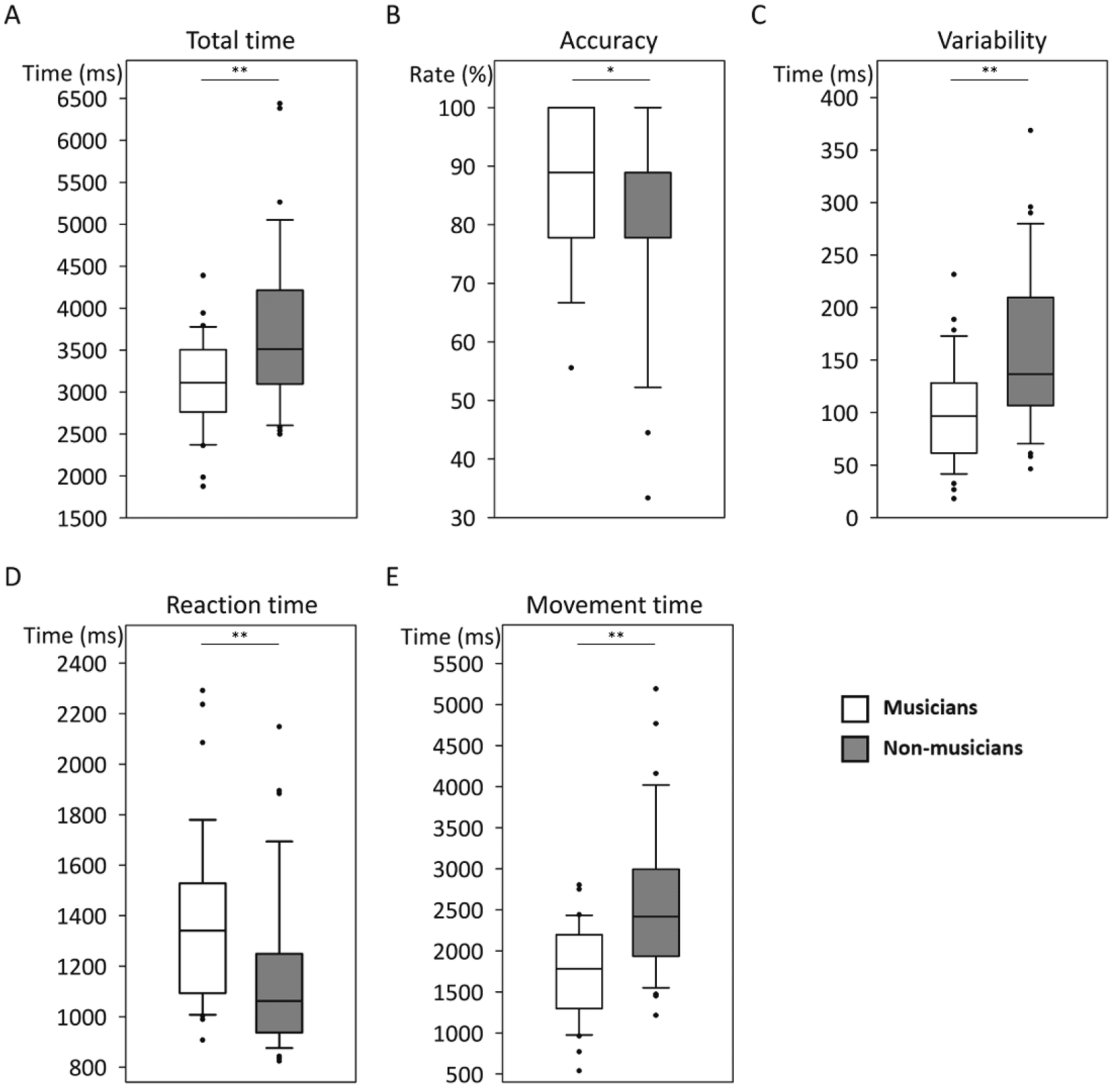
Results of the finger sequence task in musicians and non-musicians. (**A**) Total time; (**B**) Accuracy; (**C**) Variability (standard deviation, SD); (**D**) Reaction time; (**E**) Movement time. Group data are shown in box plots: white indicates musicians; gray indicates non-musicians; upper to lower limit of the box: interquartile range (IQR); whiskers above and below the box: 1.5 × IQR; middle horizontal black line: median; individual data points: values exceeding 1.5 × IQR. *p < 0.05; **p < 0.005.

For the finger sequence task (FST), musicians were significantly faster in total time (Fig. 1A) and more accurate (Fig. 1B) compared to the non-musicians. The variability of key pressing interval was significantly less in musicians than in non-musicians (Fig. 1C). Therefore, musicians performed the sequences faster and more accurately with less movement variability, compared to the non-musicians. In decomposing total time into reaction time and movement time, non-musicians demonstrated a shorter reaction time, compared to musicians (Fig. 1D). However, the musicians had significantly shorter movement time (Fig. 1E). No between-group differences were found in the Purdue pegboard test (PPT).

### Interhemispheric inhibition outcomes

**Figure 2:**
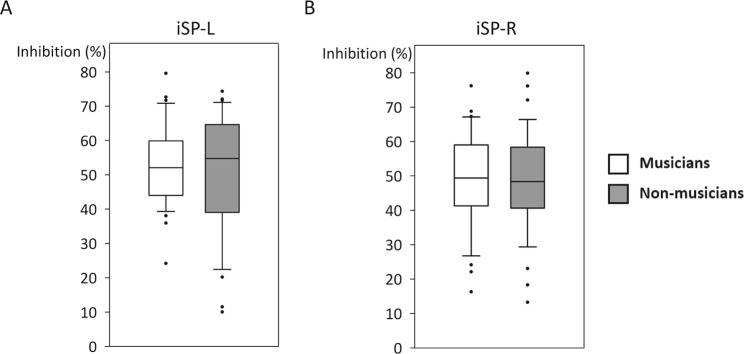
Results of the ipsilateral silent period (iSP) in musicians and non-musicians. (**A**) iSP-L: iSP measured in the left hemisphere; (**B**) iSP-R: iSP measured in the right hemisphere. Figure convention is the same as Fig. 1.

Average resting motor threshold was 52.5 ± 12.4% maximum stimulator output (MSO) in musicians and 50.3 ± 9.6% MSO in non-musicians. For the ipsilateral silent period (iSP) results, in musicians, the duration was 27.9 ± 12.3 ms in left (L) to right (R) hemisphere inhibition (iSP-L) and 27.4 ± 11.6 ms in R to L hemisphere inhibition (iSP-R); in non-musicians, the duration was 29.3 ± 7.9 ms in iSP-L and 28.5 ± 8.0 ms in iSP-R. There were no between-group differences observed in the amount of inhibition measured in either L or R hemisphere (iSP-L, Fig. 2A; iSP-R, Fig. 2B).

### Canonical correlation

**Figure 3:**
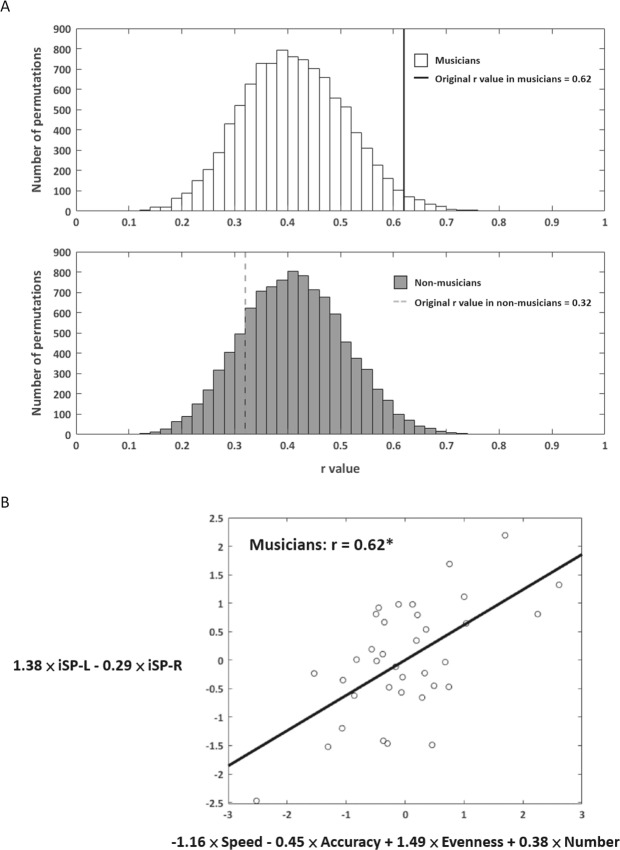
Permutation testing and the canonical relationship between interhemispheric inhibition and bimanual coordination outcomes. (**A**) Permutation testing of the correlation coefficients (r) in musicians (upper panel) and non-musicians (lower panel). X axis: r value in each permutation; y axis: number of permutation occurring in a given r value range; solid black line: original r value in musicians (r = 0.62); dashed gray line: original r value in non-musicians (r = 0.32). The chance of the permutated r values larger than the original r values was less than 5% (500 out of 10,000 times) only in musicians. (**B**) Canonical relationship in musicians. Canonical variate U: speed, accuracy, evenness, and number on the x axis. Canonical variate V: iSP-L and iSP-R on the y axis. Correlation equation: 1.38 × iSP-L − 0.29 × iSP-R = −1.16 × Speed - 0.45 × Accuracy + 1.49 × Evenness + 0.38 × Number. Significant canonical relationship was found (r = 0.62, *p* = 0.02) between bimanual coordination and IHI outcomes.

Canonical correlation analysis (CCA) generated a first order and a second order linear relationship with two IHI outcome variables and four bimanual coordination outcome variables for both groups. For the musicians, the first-order canonical correlation showed a significant IHI-bimanual coordination relationship (r = 0.62, Wilk’s lambda = 0.55, *p* = 0.02), whereas no significant relationship was observed in the non-musicians (r = 0.32, Wilk’s lambda = 0.87, *p* = 0.29). The permutation testing with 10,000 repetitions showed that there was only 193 times (1.93% chance) that the linear relationship for musicians was larger than the original r value. This indicates that the observed canonical relationship for musicians was statistically significant and was not by chance (i.e. <5% probability of making type I error) (Fig. 3A). The significant canonical correlation with corresponding canonical variates in musicians is shown in Fig. 3B. Conversely, the permutation testing showed that the chance of the permutated r values larger than the original r value was greater than 5% in non-musicians, indicating that the association was not statistically sound and could occur by chance.

Of the bimanual coordination outcomes, speed (coefficient: −1.16) and evenness (coefficient: 1.49) demonstrated larger coefficients than accuracy (coefficient: −0.45) and number (coefficient: 0.38). Thus, it appears that speed and evenness contributed to the IHI-bimanual coordination relationship to a greater extent than accuracy and number did. A multiple regression model was performed subsequently using speed and evenness as contributing behavioral outcomes to predict overall IHI (combining 1.38 × iSP-L − 0.29 × iSP-R in the y axis of the canonical correlation model). After variable reduction, speed (coefficient: −0.47, *p* = 0.084) and evenness (coefficient: 0.77, *p* < 0.01) still significantly predicted IHI (R^2^ = 0.264, *p* < 0.01), indicating that IHI is a critical modulator to maintain high speed and evenness in the FST.

## Discussion

The current study aimed to investigate whether modifications in interhemispheric communication as a function of prolonged musical training is associated with skilled bimanual coordination. We used two bimanual motor tasks to test the participants’ bimanual coordination abilities with respect to simultaneously moving fast, accurate, and even. The musicians appeared to utilize interhemispheric inhibition for modulating bimanual performance of the FST, whereas IHI was not associated with dexterous hand performance in non-musicians.

In addition to understanding the relationship between IHI and bimanual coordination, we were interested in identifying whether the communication between the two cerebral hemispheres had been modified by long-term instrument training by comparing IHI in musicians and non-musicians. Even though IHI is not measured during the actual performance of a task, it is a cross-sectional physiological observation that could be a sensitive marker to account for differences in motor behavior, such as mirror activity in EMG and force control^25,26^, or behavioral changes in response to various factors, such as aging and motor practice^27–30^. The corpus callosum, as the critical structure mediating IHI, has been shown to reorganize following instrument training with this structural reorganization correlates with musical skills^14,31^. We also asked whether bimanual coordination capability was different between the two groups. We did not find a group difference in iSP independent of the bimanual coordination measures. This result is contrary to what has been reported^16^. Chieffo *et al*., (2016) found that iSP was significantly different between musicians (all pianists) and non-musicians. While the absolute amount of IHI in both musicians and non-musicians was similar to our results, we did not detect any group differences. Given the similar iSP measurement and analysis methodology between the two studies, the explanation for the discrepant results is unclear. A larger sample size (current study: N = 72; Chieffo *et al*.: N = 30), highly skilled musicians compared to amateurs and various instrument types compared to keyboard only, both left and right hand dominant participants compared to right hand only, marked the main differences between the current study and Chieffo *et al*., (2016) respectively. For the current study, we would conclude that differences in IHI are not revealed independent of behavior.

With respect to assessment of bimanual coordination independent of IHI, we uniquely employed a task that required accurate performance of three randomly presented, 8-element sequences. Additionally, the finger sequence task used in the current study requires both temporal and spatial precision between the two hands. Previous studies used only a simple reaction time paradigm (one finger to press a button in response to a predictable stimulus as fast as possible) and showed reduced reaction time in musicians compared to non-musicians^9,32^. Using essentially a ‘choice’ reaction time paradigm that required motor planning prior to execution of movement, our data revealed a *longer* reaction time in musicians compared to non-musicians. Conversely, we found that once planned, musicians were significantly faster in performing the sequence compared to non-musicians all the while maintaining greater accuracy. It is possible that the musicians and non-musicians used different strategies to perform the FST. The musicians considered the 8-element sequence as a “whole sequence” and used more time for planning before movement initiation. Once the musicians started to move, they were able to finish the movement quickly. The non-musicians may not “chunk” the 8-element sequence into a single motor plan and thus may have spent additional time planning during movement execution. As musical training involves acquisition of various musical note sequences, the strategy adapted by the musicians for the FST may result from inherent task features that are similar to instrument playing. The distinct strategy used by the musicians demonstrating faster, more accurate movement and more consistent inter-tap interval, while spending longer time in motor planning, may result from their instrument training (e.g. planning ahead and playing the melody with consistent rhythm).

As different instruments may require distinct hand coordination skills, it is possible that the current results may have been driven by a specific instrument type(s). To address this issue, we conducted a post-hoc analysis of the bimanual coordination and IHI outcomes between the six instrument types (keyboard, percussion, woodwind, string, brass, and plucking) included in the current study. No difference was found across instrument types in any of the outcomes (independent-samples Kruskal-Wallis tests: *p* values > 0.05 in all bimanual coordination and IHI outcomes). Therefore, it was confirmed that the observed IHI-bimanual coordination relationship was independent of instrument type.

It was in the analysis of the *relationship* between IHI and bimanual coordination that unique differences between musicians and non-musicians were revealed. Canonical correlation analysis allows us to comprehensively investigate the relationship between the two IHI variables as well as the multiple measures of coordination (spatial and temporal optimization of movement speed, accuracy, and evenness)^33^. Without an a priori hypothesis that any of the variables would be more important than the others, CCA is an ideal approach to address the overall association between IHI and bimanual coordination and the weighting of each variable informs its importance in this IHI-bimanual coordination relationship. The inhibition from L to R hemisphere (i.e. iSP-L), as well as evenness and speed, were the dominant variables in the canonical relationship for musicians. The variable reduction analysis further confirmed that IHI was associated with enhanced evenness in the key pressing interval (i.e. reduced variability), with a tradeoff of slower movement speed. Of the bimanual coordination characteristics, *evenness* compared to speed and accuracy is certainly the most uncommon requirement of a motor task and possibly the most demanding. Therefore, evenness may facilitate the need for the two hemispheres to interact. However, the interhemispheric communication associated with evenness was modified by long-term musical training. We believe this may be related to better left hand performance in musicians compared to non-musicians. L to R IHI impacts the processing of information for the R hemisphere controlling the left hand. By having both groups of subjects perform a unimanual FST, we were able to demonstrate superior L hand performance in the musicians compared to the non-musicians (Supplementary Fig. 1). Perhaps greater L to R IHI enables musicians to allocate necessary resources for meeting evenness demands with the non-dominant left hand^34,35^. For non-musicians, evenness, as a goal may be so unique and challenging that greater processing is needed to solve the problem of maintaining temporal consistency between finger taps even with the dominant right hand.

We additionally examined whether the demographic variables and documented musical training-specific outcomes influenced results related to IHI, bimanual performance, and IHI-bimanual coordination relationship in musicians. Age (r = 0.53, *p* < 0.001) and total training time (r = 0.50, *p* = 0.002) were the only two demographic variables that significantly correlated to variability (due to multiple testing in correlation analyses, significance level was adjusted to *p* value < 0.005). Age also differed slightly between musicians and non-musicians, albeit marginally significant (*p* = 0.07). No correlation between IHI and demographics was found. There was no bias in age range when sampling musicians and non-musicians in the community. Although it has been shown that in older adults, IHI decreases with aging and is associated with motor performance decline^28^, the current study did not sample adults older than 57 years of age. However, when age and total training time were used as covariates in the multiple regression model with speed and evenness as contributing behavioral outcomes to predict overall IHI, the relationship remained significant. Speed (coefficient: −0.30, *p* = 0.085) and evenness (coefficient: 0.55, *p* < 0.01) still significantly predicted IHI (R^2^ = 0.263, *p* = 0.045). In other words, demographics did not alter the observed IHI-bimanual coordination relationship in musicians.

One limitation of this study is that an unequal distribution of types of instruments played limits the generalization of the current results to all kinds of musicians playing different instruments. Second, to better address the instrument-specific effect on the relationship between IHI and bimanual coordination, an asymmetric task which resembles instruments with asymmetric hand use would be necessary. The PPT employed in the current study required only temporal asymmetry of the two hands. Studies investigating bimanual coordination have utilized one hand tracking a sine wave while the other hand generates a given amount of force as a precise form of asymmetric hand use and one that would be similar to string instrument playing^36,37^. Moreover, given the relationship between IHI and evenness as a specific feature only seen in musicians, the benefits of musical training in temporal control and rhythmicity on cognitive function is a worthwhile future investigation^38,39^.

In conclusion, the relationship between IHI and bimanual coordination in musicians appears to be altered following long-term instrument training compared to non-musicians. The interhemispheric communication may be a vehicle in the central nervous system that enables the trained musician to achieve the high skill demand essential for instrument playing. Increased inhibitory processing from the L to R hemisphere accounts for reduced movement variability, possibly through activating the R hemisphere for more refined control of the L hand. Utilizing this IHI-dependent strategy, greater movement consistency is achieved while speed is modulated. Experience-dependent modulation of the communication between bilateral hemispheres result from extensive musical training cannot be revealed independent of behavior.

## Materials and Methods

### Participants

Thirty-six musicians (keyboard: 14, percussion: 1, woodwind: 7, string: 8, brass: 2, plucking: 4) and 36 age-matched non-musician controls participated in this study. The musicians were classically-trained professionals or music-major college students who regularly and intensively practiced musical instruments. All of the musicians had been practicing and performing since early childhood. The age-matched non-musicians were not engaged in any intensive fine motor activities for more than 1 hour per day (e.g. intensively typing, texting on cell phones, or video game playing). The participants were screened using a transcranial magnetic stimulation (TMS) safety questionnaire and were excluded if there were any contraindications to TMS procedures^40^. Handedness was measured by the Edinburgh Handedness Inventory^41^. Musical skills and behaviors on multiple dimensions were measured by the Goldsmiths Musical Sophistication Index^42^. The current study was approved by the Institutional Review Board of the University of Southern California and informed consent was obtained from each participant. All study protocols were performed in accordance with guidelines and regulations of the University of Southern California.

### Bimanual coordination assessment

#### Finger sequence task (FST)

**Figure 4:**
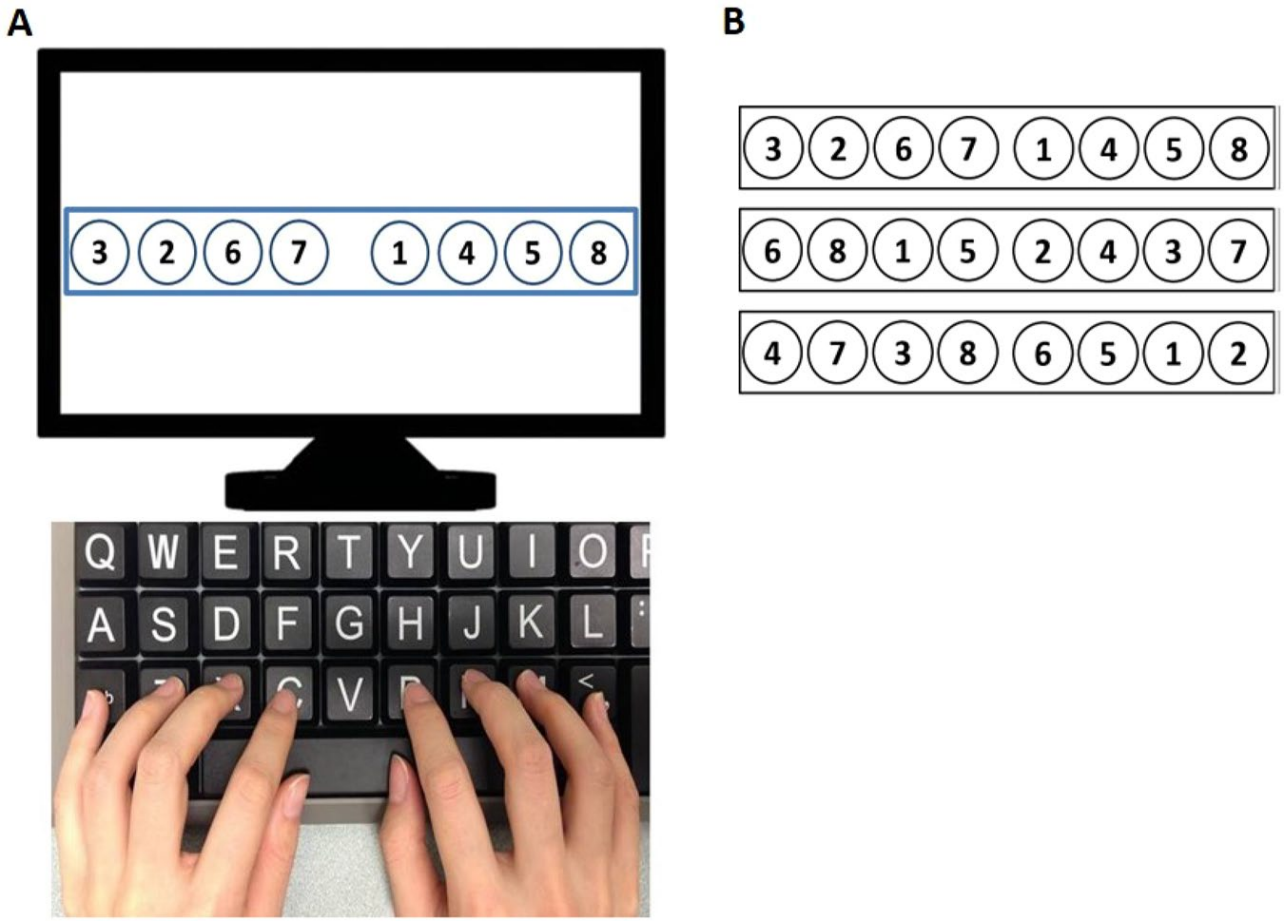
Finger sequence task. (**A**) Experimental setup. Participants were instructed to put the index, middle, ring and little fingers of both hands on the designated keys of an enlarged computer keyboard. Each 8-element sequence was shown on the computer screen and the participants pressed the corresponding keys in a sequential order as fast, accurately, and evenly as possible. (**B**) Three sequences used in the assessment.

For testing symmetric hand coordination and agility^43,44^. The participants performed an 8-element sequence of finger movements on a computer keyboard (Fig. 4A). Participants were instructed to press the keys in an ascending order: 1-2-3-4-5-6-7-8. Three sets of sequences (Fig. 4B) were used and the participants had 10 minutes to practice all of the assigned sequences before the start of the test. The practice was divided into two parts. For the first 5 minutes, three sequences were provided on the computer screen at the same time. Participants were instructed to practice all three sequences (in their own strategies, without feedback) and to familiarize themselves with the three sequences. For the last 5 minutes, participants practiced the sequences in the real testing interface, with one of the three sequences randomly presented on the computer screen in each trial, and their performance was recorded to provide feedback. The participants were asked to perform the FST as fast and as accurately as possible while maintaining an even key pressing interval (i.e. reduce variability). In other words, the task goals were to maximize **speed**, **accuracy**, and **evenness**. In the testing, the three sequences were presented in a pseudorandom order and there were a total of nine trials (3 trials per sequence). Feedback in the form of time to complete each sequence and the accuracy of the sequence were provided at the end of each trial. Total time (task completion time), accuracy, and variability of key pressing interval (the standard deviation (SD) of the time difference between two consecutive key presses) were recorded as motor performance. Total time was further decomposed into reaction time (time between the presentation of the sequence and first key press) and movement time (time between the first and final key press) to determine movement planning and movement execution, respectively. The investigator checked participants’ performance and made sure they understood the task goals and could perform all three sequences as fast, as accurate, and as even as possible.

#### Purdue pegboard test (PPT)

**Figure 5:**
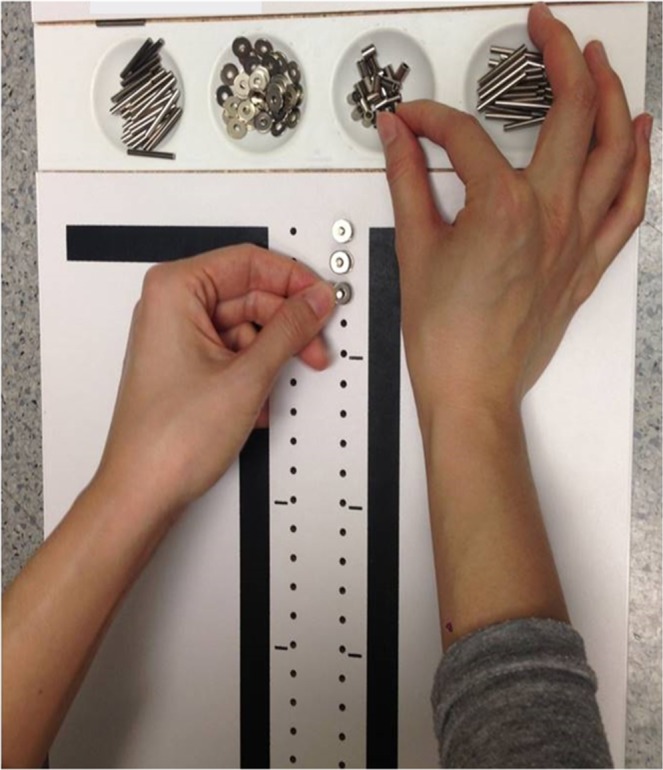
Purdue pegboard task. A pegboard was placed in front of the participants; they used both hands to pick up the objects (pins, washers, and collars) in the cups and alternately assembled the objects into the holes embedded in the pegboard. The designated sequence was pin, washer, collar, and washer, starting with the dominant hand.

For testing hand dexterity during performance of an asymmetric task^45^. The PPT is a bimanual coordination assessment tool using a functional daily activity (picking up small objects) and is widely used in clinical settings and research. This is a task in which small objects are picked up by both hands and are placed consecutively into holes embedded in a pegboard. There are three types of objects (pins, washers, and collars), which the participants pick up from different cups. Participants were instructed to assemble the objects into an identical hole using the two hands alternately in one minute. The sequence was pin, washer, collar, and washer (Fig. 5), with one hand grasping one object at a time. The **number** of objects successfully put into the holes was the motor performance outcome.

### Interhemispheric inhibition assessment

Ipsilateral silent period was measured by TMS to index interhemispheric inhibition. The methods used for iSP acquisition and quantification were identical to those detailed in our previous study^46^. First, the maximum voluntary contraction (MVC) of the abductor pollicis brevis (APB) for both hands was measured. APB was chosen as the target muscle given that this muscle yields a stable and apparent onset and offset of electromyography (EMG) silence, and thus consistent iSP results^47^. The participants were instructed to abduct the thumb to 50% of MVC while a single TMS pulse (intensity: 130% resting motor threshold) was applied to the representational area of the APB in the ipsilateral primary motor cortex. Online biofeedback was provided to ensure that participants maintained isometric thumb abduction throughout each trial before the application of the TMS. The temporary reduction of muscle activity recorded by EMG observed in the contracting thumb is termed ipsilateral silent period. Fifteen trials were obtained for the L hemisphere APB representational area with L hand activation and 15 trials were obtained for the R hemisphere APB representational area with R hand activation. Matlab (The MathWorks Inc., Natick, MA, USA) was the data analysis software used to process raw EMG data and determine iSP onset, offset, and duration using an objective graphical method^46,48,49^. Onset was determined as the data point fell below the variation limit of two standard deviation and offset was determined as the EMG trace reached back to the baseline. Duration was calculated as the difference between onset and offset. Ipsilateral silent period was quantified as the amount of EMG reduction in the determined iSP duration following TMS, normalized to pre-stimulation muscle contraction level. The iSP measurement was quantified as the percentage of decreased muscle activity indicating the amount of inhibition from the stimulated hemisphere. This quantification method was selected based on the inherent small measurement variability^46^. For delineating the results of the study, iSP-L is indicative of L to R inhibition and iSP-R indicative of R to L inhibition^16^.

### Statistical analyses

Independent *t* tests were used to compare differences in bimanual coordination outcomes and differences in IHI outcomes between musicians and non-musicians. Canonical correlation analysis was used to identify a linear relationship between combinations of multiple variables (bimanual coordination outcomes and IHI outcomes). As part of the analysis each variable was weighted, demonstrating the magnitude and direction of the contribution of each outcome in the linear relationship^50–52^. All of the bimanual coordination and IHI outcomes for all participants were converted into z scores to allow unit-less comparisons across different measures. The bimanual coordination outcomes included: speed (total time × (−1)), accuracy, and evenness (variability × (−1)) for the FST, and number for the PPT, such that higher z scores indicated better motor performance. Only total time was included in the CCA as it best reflects the instructions given to the participants as well as to minimize the number of variables introduced. Speed and evenness z scores were multiplied by minus one in order to make the direction of change in all variables consistent (higher values indicated better motor performance). The IHI outcomes included iSP-L and iSP-R, with higher z scores indicating more IHI. Two canonical correlation analyses were performed with 36 musicians and with 36 non-musicians’ z scores. A canonical variate U (a weighted bimanual coordination score) and a canonical variate V (a weighted IHI score) for each participant within the two groups were used to calculate the correlation between all U and V values in each group.

To validate whether the strength of the linear relationship between the canonical variates was not by chance (i.e. r values significantly different from zero), permutation testing was performed for each group. The z values were randomly re-assigned to each participant and the same CCA procedure was repeated 10,000 times. Validation of the original r value for each group occurred as follows: the number of times that the r value after random re-assignment of the z scores exceeded the original r value was determined (10,000 r values compared to the original r value). If the permutated correlation coefficients were equal to or larger than the original r value less than 500 out of 10,000 times, then the probability of the original correlation coefficient occurring by chance was less than 5% (i.e. two-tailed *p* value < 0.05), indicating a statistically significant correlation in the original linear relationship.

Following the results and validation of the CCA, a multiple regression analysis was performed in variables with higher weightings in the canonical relationship to confirm their explanatory power in the brain-behavior relationship.

## Supplementary information

Dataset 1

## Data Availability

De-identified data are available upon reasonable request.
