# Supplementary material for: Relationship between Interhemispheric Inhibition and Dexterous Hand Performance in Musicians and Non-musicians
Source: Sci Rep. 2019 Aug 9;9:11574. doi: 10.1038/s41598-019-47959-y (PMC6689014; doi:10.1038/s41598-019-47959-y)
Supplement: Supplementary file 1 — Dataset 1 [file 41598_2019_47959_MOESM1_ESM.pdf]

Supplementary Information

Relationship between Interhemispheric Inhibition and Dexterous Hand Performance in Musicians and Non-musicians

Yi-Ling Kuo[1,2], Jason J. Kutch[1,3], Beth E. Fisher[1,4 *]

1 Division of Biokinesiology and Physical Therapy, University of Southern California, Los Angeles, CA

2 Massachusetts General Hospital Institute of Health Professions, Boston, MA

3 Neuroscience Graduate Program, University of Southern California, Los Angeles, CA

4 Department of Neurology, Keck School of Medicine, University of Southern California, Los Angeles, CA

Corresponding author: Beth E. Fisher

Address: 1540 East Alcazar Street, Room 210E, Los Angeles, CA 90089-9006

Tel: 323-442-2796

Fax: 323-442-3366

E-mail: bfisher@usc.edu

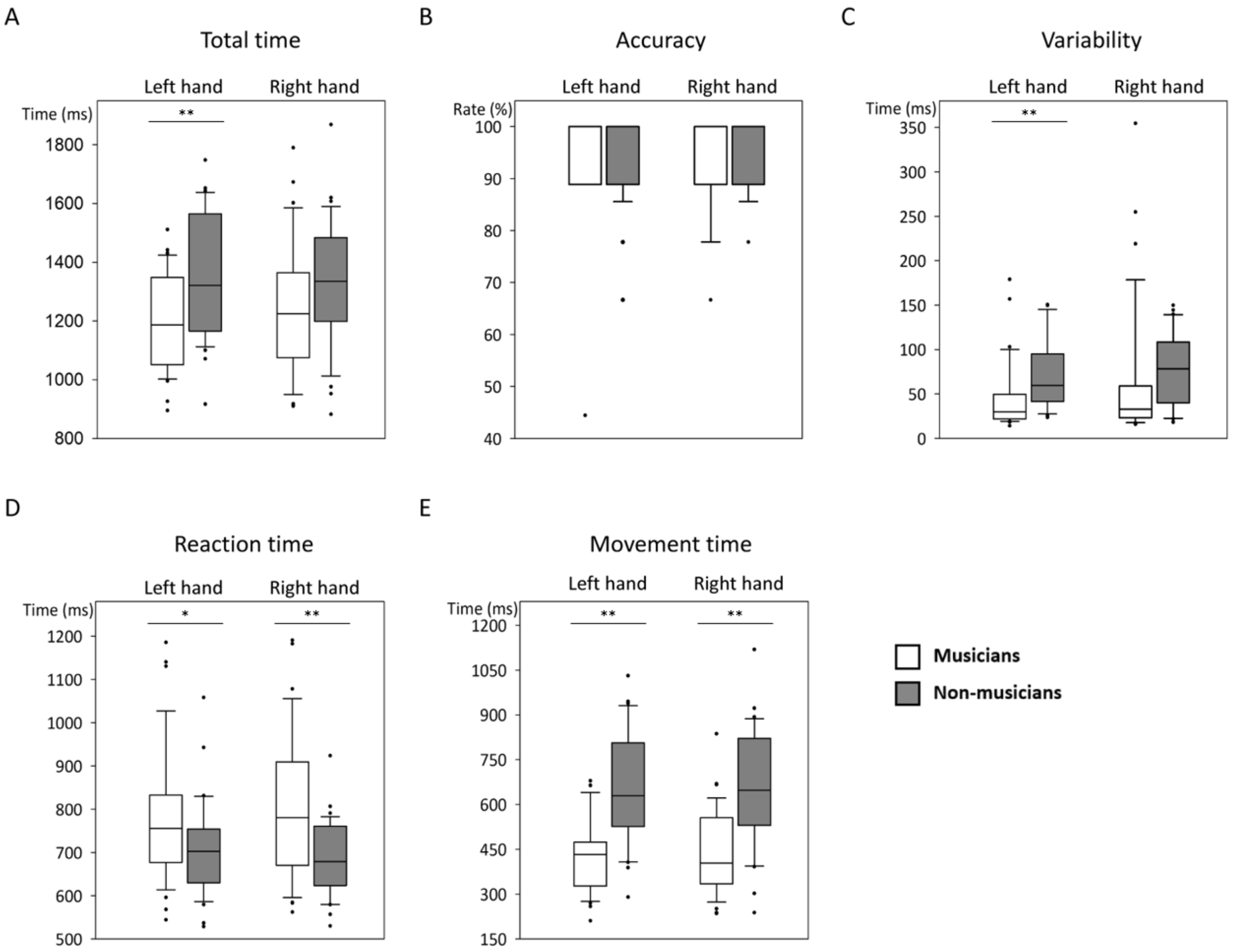

Supplementary figure 1. Results of the unimanual finger sequence task in musicians and non-musicians. A: Total time; B: Accuracy; C: Variability (standard deviation, SD); D: Reaction time; E: Movement time. Group data are shown in box plots: white indicates musicians; gray indicates non-musicians; upper to lower limit of the box: interquartile range (IQR); whiskers above and below the box: 1.5 × IQR; middle horizontal black line: median; individual data points: values exceeding 1.5 × IQR. * $p < 0.05$; ** $p < 0.005$.
